# Minimally invasive resection of cervical schwannoma (C1–C2 level) using tubular retractors

**DOI:** 10.3171/2023.7.FOCVID2286

**Published:** 2023-10-01

**Authors:** Harsh Deora, Madhusudhan Nagesh

**Affiliations:** Department of Neurosurgery, National Institute of Mental Health and Neurosciences (NIMHANS), Bangalore, India

**Keywords:** schwannoma, cervical spine, minimally invasive spine surgery

## Abstract

Cervical schwannoma excision usually involves laminectomy and violation of the facet joints that necessitates the need for fusion with consequent loss of cervical mobility. The authors present the first video demonstration of an excision of the cervical schwannoma at the C1–2 level using minimally invasive spine tubular retractors, which allows direct access to the lesion and prevents the need for any bony removal. It also preserves the paraspinal muscles, which are important for spinal stability, especially at the C2 level. Special attention is given to incision planning and preoperative image analysis for preventing injury to critical neurovascular structures at this level.

## Transcript

### 0:25 Case Description.

A 35-year-old female presented with weakness and tightness of all four limbs since 8 months and urinary urgency since 4 months. On examination, she had spasticity of all four limbs with exaggerated reflexes and motor power of 4/5.

### 0:48 MRI Contrast Sagittal and Axial Cuts.

Magnetic resonance contrast-enhanced images showed an extradural well-defined enhancing lesion at the C1–2 level with severe compression of the cervical cord medially. The lesion extends to the cervical foramina with the vertebral artery seen anteriorly.

### 1:19 Treatment Plan.

While traditional planning would dictate midline incision and C1–2 laminectomy and excision of the lesion with fixation using screws and rods, we preferred a more elegant parasagittal approach. This allows for direct access to the lesion and prevents the need for any bony removal or paraspinal muscle damage.^1,2^

### 1:41 Positioning and Placement of Monitoring Electrodes.

The patient was taken to the operating room and placed prone, and the head fixed in the neutral position using Mayfield three-pin head holder. Motor evoked potential (MEP) monitoring electrodes were placed, and the recordings remained baseline throughout the procedure. No triggered EMG was used as C2 nerve root does not have any motor supply.^3^

### 1:45 Incision and Docking.

The incision was placed 2 cm lateral to the midline and level confirmed using a lateral x-ray. Subsequently, the tubular dilators were placed serially and the expandable tube was docked and expanded. The tube was 4 cm in length and 20 mm in diameter, which after expansion led to a 30-mm diameter.

### 2:12 Post Exposure View.

As soon as the tubular retractors were docked and expanded, the C1–2 lamina and schwannoma was seen clearly and the soft tissue over the same was dissected off.

### 2:3 Capsule Opening.

The capsule was then opened in a cruciate manner and the schwannoma is seen as a glistening white structure below it.

### 2:59 Surgical Technique.

Following this, the schwannoma was circumferentially dissected off the capsule and internal decompression was done using the Cavitron ultrasonic aspirator. The blunt hook was then used to roll the lesion in the operative field and further decompression was done. Once adequate decompression was achieved, the schwannoma was then delivered en bloc, taking care not to damage the dura medially or the vertebral artery anteriorly. It is important to dissect all around the schwannoma before attempting to completely remove the tumor. Decompression of the tumor allows the tumor to be rolled within the capsule. Only when the tumor is completely mobile should an en bloc removal be attempted. The tubular retractor gives good anterior access from this route, and with adequate precautions taken, vertebral artery injury can be avoided. However, in the rare case of such a catastrophic event, a midline rescue incision has been shown and the paramedian incision can be fashioned in a curvilinear manner to allow for greater exposure.

### 4:42 Capsule Dissection of C2 Nerve Root.

The capsule was then carefully dissected of the C2 nerve root using a combination of gentle bipolar coagulation, sharp dissection, and traction-countertraction. If the schwannoma arises from one rootlet of the cervical nerve with a large exophytic component, as in this case, resection of the same without complete sectioning of the root is possible.^4^ Then the dura was completely decompressed and pulsating well.

### 5:20 Closure.

The brisk venous bleeding was then controlled using Gelfoam packing, and tissue sealant was then used as a preventive measure to prevent cerebrospinal fluid leak. As mentioned, one of the rootlets may have been inadvertently damaged or resected during removal of the capsule, which could lead to CSF leak at a later date. Hence, as a precautionary measure fibrin glue was used. The tubular retractors were then removed, and the muscle collapsed completely and was closed in a standard three-layer fashion.

### 5:48 Follow-Up.

This incision healed well at follow-up and magnetic resonance imaging at 1-year follow-up showed no residue of the lesion with the parasagittal route seen. Preoperative signal change had reduced in T2 sequences as compared to preoperative images; however, they remained at 1-year follow-up imaging as well. Postoperative signal reduction has been shown to be associated with better clinical outcomes and symptomatic improvement. Persistence of the same or an increase leads to persistence of troubling symptoms such as paresthesia and spasticity.^5^ This patient had a remarkable improvement in spasticity and power in all four limbs, and at 1-year follow-up there were no more residual symptoms.

### 6:00 MRI T2 Sagittal and Axial Cuts.

Follow-up at 12 months shows no residue, with the cervical cord now centrally placed and decompressed completely. This procedure and route allows for a complete and safe resection of cervical schwannoma with an excellent cosmetic result and prevents the need for any fusion procedure or paraspinal muscle dissection.^3^

## Disclosures

The authors report no conflict of interest concerning the materials or methods used in this study or the findings specified in this publication.

## Author Contributions

Primary surgeon: Deora. Assistant surgeon: Nagesh. Editing and drafting the video and abstract: both authors. Critically revising the work: Deora. Reviewed submitted version of the work: Deora. Approved the final version of the work on behalf of both authors: Deora. Supervision: Deora.

## Supplemental Information

### Patient Informed Consent

The necessary patient informed consent was obtained in this study.
